# First-Principles Studies of Adsorptive Remediation of Water and Air Pollutants Using Two-Dimensional MXene Materials

**DOI:** 10.3390/ma11112281

**Published:** 2018-11-14

**Authors:** Yujuan Zhang, Ningning Zhang, Changchun Ge

**Affiliations:** School of Materials Science and Engineering, University of Science and Technology Beijing, Beijing 100083, China; zhangningning.1211@163.com

**Keywords:** MXene, first-principles, adsorption, environmental remediation

## Abstract

Water and air pollution is a critical issue across the whole world. Two-dimensional transition metal carbide/nitride (MXene) materials, due to the characteristics of large specific surface area, hydrophilic nature and abundant highly active surficial sites, are able to adsorb a variety of environmental pollutants, and thus can be used for environmental remediation. First-principles method is a powerful tool to investigate and predict the properties of low-dimensional materials, which can save a large amount of experimental costs and accelerate the research progress. In this review, we summarize the recent research progresses of the MXene materials in the adsorptive remediation of environmental pollutants in polluted water and air using first-principles simulations, and try to predict the research direction of MXenes in the adsorptive environmental applications from first-principles view.

## 1. Introduction

Water and air pollution has been always a critical issue across the whole world. The pollutants exist in a variety of forms, including heavy metal ions, toxic organics, gases, bacterium, and even radionuclides. They can pose significant negative effects on human beings and other living organism. For example, in some cases, they cause serious diseases, and even cancer, to human beings [1]. Among various techniques for the removal of pollutants, such as membrane filtration, precipitation, solvent extraction and ion exchange [2,3,4,5], adsorption especially attracts people’s attention, since it possesses several advantages, e.g., easy-operation, cost-effective, and also can avoid secondary pollution by generating other harmful substances [6,7,8,9]. The adsorbents usually have large specific surface area, and active functionalities for pollutants. Nowadays, activated carbon has been widely used for the removal of industrially discharged pollutants [10].

Since graphene was discovered [11], two-dimensional (2D) materials have been garnering great attentions due to the unique properties different from their bulk counterparts. After graphene, more and more members of the 2D family have been discovered, such as transition metal dichalcogenides (TMD), hexagonal boron nitrides, phosphorenes, etc. [12,13,14]. These materials have been demonstrated to be of promising applications in diverse areas. In 2011, Ti_3_C_2_ layered materials were first synthesized using a hydrofluoric acid (HF) etching process [15]. In the years afterward, more 2D transition metal carbide/nitride (labeled as MXene) materials were synthesized [16,17,18]. MXenes usually combine the properties of both metals and ceramics, such as high chemical stabilities and high electrical conductivities, behaving as “conductive clays” [19]. MXenes have great potential applications in the energy-storage area, e.g., supercapacitors, and lithium-ion batteries [20,21,22]. The as-prepared MXenes using chemical etching method generally possess hydrophilic nature and abundant highly active functional groups on the surfaces, and therefore are able to effectively adsorb various pollutants. 

Experimental studies are usually subject to many factors, e.g., materials, equipment, and costs, and therefore limit the research speed. However, theoretical simulation such as density functional theory (DFT) based first-principles method is an effective approach to analyze and predict the properties of low-dimensional materials. For the studies of the adsorption of pollutants on MXenes, first-principles method can help researchers better predict the promising MXene candidates that can efficiently adsorb specific toxic pollutants, thereby, eliminating the need to conduct experimental studies with unproductive outcomes. Thus, in this regard, first-principles methods can help save time and resources, but give very convincing results. 

In this paper, we review the recent research progress of the MXene materials in the adsorptive remediation of environmental pollutants in polluted water and air using first-principles simulations, including heavy metal elements, radionuclides, and gaseous molecules, and try to predict the research direction of MXenes in the adsorptive environmental applications based on first-principles theory. We hope this review can help the theoretical researchers widen their research area, and even accelerate the pace of applying the MXene materials in practical environmental systems.

## 2. Brief Introductions to the Development of First-Principles Simulation

Density functional theory-based first-principles simulation is a quantum mechanical method that obtains electronic structures of materials through resolving Schrödinger equation and further predicts the physical properties of the materials. This method starts from five basic physical constants, i.e., electron mass, electron charge, Planck constant, speed of light, and Boltzmann constant, and does not rely on any empirical constants, and thereby considered as a very accurate approach. With the development of high-throughput computing science, first-principles calculation method has achieved significant development and been widely used to study the physicochemical properties of materials.

The theoretical study of strong correlation systems like transition metals, lanthanides, and actinides-including systems, involves relativistic effects and strong electronic correlation effects that cannot be dealt with by traditional density functional theory. For example, Pacchioni et al. have proved that the adsorption properties of CO on the Pt (100) surface can be well explained by taking into account the scalar relativistic effects [23]. The scalar relativistic effect containing the relativistic mass shift and the Darwin term is included in the projector augmented wave pseudopotential in the studies of uranium ion adsorption on MXene materials by Zhang et al. [24,25] In recent years, breakthroughs in the calculation of strong-correlation materials based on density functional theory have been made, such as hybrid density functional method [26], self-consistent field interaction correction [27] and density functional theory plus *U* (DFT+U) method [28]. The above methods correct the strong correlation between 5*f* electrons, making it possible to accurately simulate strong-correlation systems. Hybrid density functional method introduces the non-local Hartree-Fock exchange term in the correlation term, which corrects the deficiency of the traditional density functional theory method in dealing with self-interactions [26]. This method can accurately describe the structures, density of states, and optical band gap properties of insulating materials. Wen et al. accurately described the electronic and structural properties of lanthanide dioxide using the hybrid density functional method [26]. Self-consistent field interaction correction can be regarded as a generalized Hartree–Fock approximation considering the dynamic Coulomb shielding interaction [27]. It is used to eliminate the unreal electronic self-interactions in the band structure theory derived from the local spin density approximation (LSDA). Petit et al. applied this method to the calculation of the ground state electronic structure of the lanthanide nitride [29]. The DFT+U method adds a Hubbard *U* term to the Hamiltonian, which can correct the strong correlation interaction between electrons. The DFT+U method has been developed into an effective theoretical research tool in dealing with strongly correlated electronic materials. Guo et al. have studied the adsorption properties of Pb and Cu on MXenes using DFT+U method [30]. For the metastable problem in DFT+U calculations, different approaches such as occupation matrix control (OMC) method [31], U-ramping method [32], quasi-annealing (QA) method [33] and controlled symmetry reduction (CSR) method [28,34] have been developed. 

In addition, the first-principles dynamics method combining the first-principles method for solving the Schrödinger equation and the classical molecular dynamics method has gradually become an important theoretical research tool for the simulation of dynamic processes in materials. The biggest difference between the first-principles dynamics method and the classical molecular dynamics method is the way to obtain the interaction between particles. The force in the classical molecular dynamics method is obtained by the potential function, and the first-principle dynamics method is based on the electronic wave function, and the force between the particles is derived by the Hellmann–Feynman theorem [35].

## 3. Structures of MXene Materials

**Figure 1:**
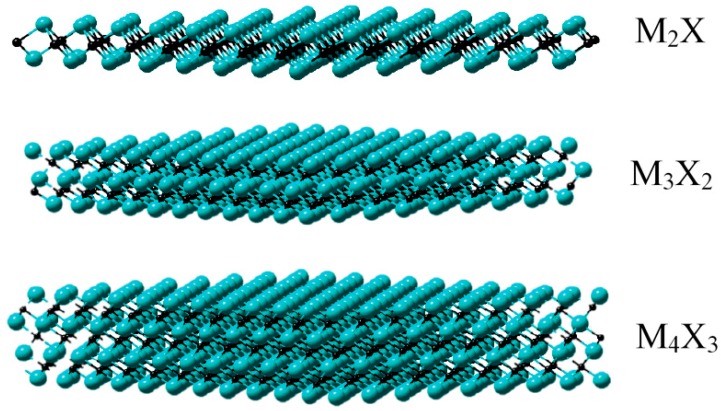
The early reported three different structures of 2D transition metal carbide/nitride (MXenes) (non-terminated): M_2_X, M_3_X_2_ and M_4_X_3_. Reprinted from Ref. [22] with permission. Copyright 2017 Macmillan Publishers Limited. (Color online).

Typically, MXenes can be synthesized from “MAX” matrix phase using a chemical etching process, e.g., hydrofluoric acid (HF), hydrochloric acid (HCl) combined with lithium fluoride (LiF) [17,19]. The term “MAX” represents the reactant compounds M*_n_*_+1_AX*_n_* (*n* = 1, 2, 3), where M denotes a transition metal element, A stands for a group IIIA or IVA element (A = Al, Ga, In, Si, Ge, Sn, Pb, P, As, S and Cd) and X is C or N element. An example of MAX is Ti_3_AlC_2_, from which the first MXene material, Ti_3_C_2_, was fabricated [15]. Therefore, the structure of a MXene can be described as *n*+1 layers of M atoms covering *n* layers of X atoms in an (MX)*_n_*M arrangement. So far, at least three different MXenes, M_2_X, M_3_X_2_ and M_4_X_3_, have been confirmed [17,22], as shown in Figure 1.

**Figure 2:**
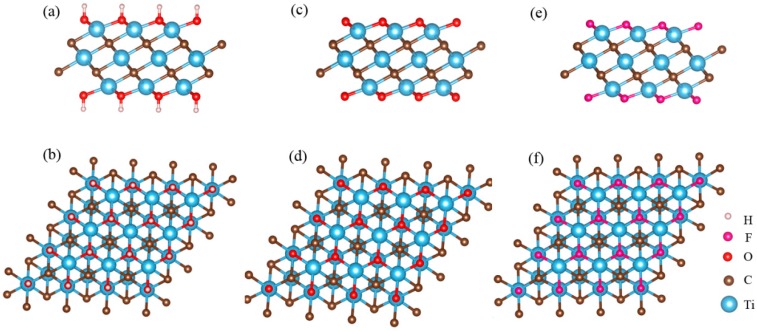
The structure of Ti_3_C_2_ nanosheets with different functional groups from side and top views: (**a**,**b**) Ti_3_C_2_(OH)_2_; (**c**,**d**) Ti_3_C_2_O_2_ and (**e**,**f**) Ti_3_C_2_F_2_. (Color online).

MXenes synthesized from chemical etching method usually possess various surficial functional groups, the most common ones of which are oxygen (–O), hydroxyl (–OH) or fluorine (–F) [36,37,38]. Therefore, the chemical formula of MXene is generally written as “M*_n_*_+1_X*_n_*T_x_”, where T denotes the surficial functional groups. For example, Ti_3_C_2_ MXene synthesized from chemical etching method can at least have the following three formulae: Ti_3_C_2_(OH)_2_, Ti_3_C_2_O_2_ and Ti_3_C_2_F_2_, as shown in Figure 2. The species and quantities of these terminal groups are highly dependent on the synthesis process. As is known, hydrophilic nature facilitates adsorption for polar or ionic pollutants, i.e, the –F group is unfavorable for adsorption, which will be discussed in the following part. 

Employing chemical vapor deposition method, MXenes without surficial functional groups have also been successfully synthesized, such as Mo_2_C, WC, and TaC [39]. Since the exposed unterminated metal atoms are highly reactive, these MXenes are prone to combining with other substances, and thus very suitable to be adsorbents. First-principles calculations have confirmed this characteristic, which will be seen in the following text. 

## 4. First-Principles Progress of Adsorption of Pollutants on MXenes

Subject to calculation resources, first-principles method is more suitable for studying the adsorption behaviors of small molecular adsorbates on MXenes, but is difficult for large and complex species. For example, experiments have confirmed the excellent adsorption behaviors of organic dyes [40,41,42,43] or even bacterium [44,45,46] on MXenes. However, first-principles based theoretical studies for the adsorption behaviors of these complex systems on MXenes are not yet reported.

According to the types of the pollutants adsorbates in water and air reported in literature, we will make discussions on heavy metal elements, radionuclides, and gaseous molecules.

### 4.1. Heavy Metal Elements Adsorption

Heavy metal elements can exist in cationic, anionic and electrically neutral forms. Heavy metal ions generally exist in water bodies, such as rivers or lakes. They can cause serious threat to human beings and other animals. Typical toxic heavy metal elements include Pb, Cr, Hg, Cd and Cu. World Health Organization (WHO) has made clear statements for the upper limits of heavy metal elements in drinking water, called WHO’s Drinking Water Standards (set up in Geneva in 1993). For example, the upper limit of Pb in drinking water is 10 μg/L, and the upper limit of Cd is 3 μg/L. Many researchers have studied the adsorption behaviors of heavy metal elements on MXenes using first-principles method, which agree well with the experimental results.

Pb(II) is the first heavy metal ion confirmed to be effectively adsorbed on MXenes by first-principles method. Peng et al. reported that NaOH-treated Ti_3_C_2_ MXene material Ti_3_C_2_(OH/ONa)_x_F_2−x_ has excellent adsorptive ability for Pb(II), and interpreted it based on first-principles theory [47]. The adsorption chemical equations are:

Ti_3_C_2_(OH)_2_ + *m* Pb(NO_3_)_2_→Ti_3_C_2_(O_2_H_2−2*m*_Pb*_m_*) + 2*m* HNO_3_

**Figure 3:**
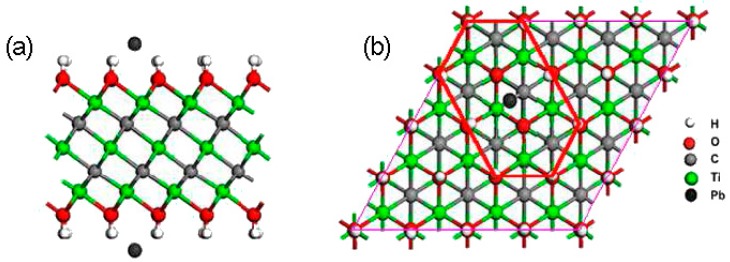
The sketch map of Ti_3_C_2_(O_2_H_2−2*m*_Pb_m_) after Pb atom replaces two H atoms: (**a**) the side view; (**b**) the top view. Reprinted from Ref. [47] with permission. Copyright 2014 American Chemical Society. (Color online).

Ti_3_C_2_(ONa)_2_ + *m* Pb(NO_3_)_2_→Ti_3_C_2_(O_2_H_2−2*m*_Pb*_m_*) + 2*m* NaNO_3_

i.e., ion exchange reactions occur between Pb and H/Na atoms. Electron localization function (ELF) calculations show that the mechanism of adsorption includes two aspects: Pb atom forms strong chemical bonds with oxygen atoms (hydroxyls losing H atoms), and at the same time with the surrounding hydroxyl groups, as in shown in Figure 3. This mechanism can be vividly described as the Pb atom being trapped by two underneath oxygen atoms and eight surrounding hydroxyl groups. First-principles calculation also predicts that the sites of hydroxyl groups and the types of surficial functional groups significantly affect the adsorption behaviors of Pb(II) on Ti_3_C_2_(OH)_2_ MXene [48]. The hydroxyl groups on top of the titanium atoms have larger adsorption energies than other adsorbed structures, implying stronger ability for the adsorption of Pb(II). Surficial fluorine groups have negative effects on the adsorption efficiency, while the addition of Li, Na, and K atoms facilitates the adsorption. Moreover, the coverage of Pb(II) on the MXene surface has important effects on the adsorption energy: the adsorption energy decreases with increasing the coverage, i.e., with increasing the adsorbed Pb atoms, further adsorption ability of the MXene is weakened. When the coverage is smaller than 1/9 monolayer (ML), the MXene exhibits strong adsorption ability for Pb(II) ions (adsorption energy greater than −1 eV). Peng et al. have also studied the adsorption behaviors of Pb(II) on different MXenes with a general form M_2_X(OH)_2_ (M = Sc, Ti, V, Cr, Zr, Nb, Mo, Hf, Ta, and X = C or N) using first-principles theory [49]. The results indicate that only Zr_2_C(O_2_H_2−2*x*_Pb*_x_*) and Sc_2_C(O_2_H_2−2*x*_Pb*_x_*) have positive formation energies, i.e., Sc_2_C(OH)_2_ and Zr_2_C(OH)_2_ MXenes are not suitable for Pb(II) removal. All adsorption products M_2_N(O_2_H_2−2*x*_Pb*_x_*) have negative formation energies, and more negative than their carbide counterparts, indicating the M_2_N(OH)_2_ MXene has stronger Pb adsorption ability than M_2_C(OH)_2_ MXene. These results are of great significance for guiding practical applications.

Similar to Pb(II), first-principles calculations show that alkaline intercalated Ti_3_C_2_ MXene can also effectively adsorb a series of other divalent heavy metal ions including Cu, Zn, Pd, Cd [48]. The adsorption chemical reaction of divalent heavy metals Y (in nitrate form) can be written as Ti_3_C_2_(OH)_2_ + *m* Y(NO_3_)_2_→Ti_3_C_2_(O_2_H_2−2*m*_Y*_m_*)_2_ + 2*m* HNO_3_. When the coverage is smaller than 1/9 mololayer (ML), adsorption energies for all four heavy metal elements are greater than −1 eV, i.e., all the ions can be effectively adsorbed on Ti_3_C_2_(OH)_2_ MXene. Ti_3_C_2_T_x_ MXene has been experimentally confirmed to be an effective adsorbent for Cu(II) ion [50]. Additionally, it is also experimentally reported that Ti_3_C_2_T_x_ MXene can effectively adsorb Ba(II) ion [51,52], yet lack of first-principles analysis. 

Except for the above positively charged heavy metal ions, MXenes have also been confirmed to adsorb negatively charged heavy metal ions. For example, experimental studies have shown that Ti_3_C_2_T_x_ MXene possesses a high purification capacity for Cr_2_O_7_^2−^ ions in water [53,54]. At low pH, the adsorption of Cr (VI) on Ti_3_C_2_T_x_ is attributed to the electrostatic attraction between positively charged surface of MXene and negatively charged Cr_2_O_7_^2−^ ion. Along with the adsorption, the MXene can reduce Cr (IV) to Cr (III) at the same time. However, there is no related first-principles analysis regarding the adsorption of negatively charged heavy metal ions on MXenes yet.

Besides the charged heavy metal ions, MXenes have also been shown to be effective adsorbents for free non-ionic heavy metal atoms based on first-principles theory. Guo et al. have systematically investigated the adsorption behaviors of non-ionic Pb and Cu atoms on different MXenes using DFT+U method, including Ti_3_C_2_, V_2_C_1_ and Ti_2_C_1_ MXenes with bare, H, OH, and F terminations [30]. The results show that surface terminations significantly influence the adsorption ability for Pb and Cu atoms. All the MXenes can effectively adsorb Pb atoms with binding energy larger than 1 eV except F terminated ones, and only bare and OH terminated MXenes can effectively adsorb Cu atoms. The mechanism of the adsorption is related to the complex interactions between the adatoms (Pb and Cu) and MXene atoms. Based on first-principles calculations, Ti_2_C(OH)_2_ and Ti_3_C_2_(OH)_2_ MXenes are also shown to effectively adsorb free non-ionic Au atoms with adsorption energy greater than 3 eV [55]. The replacement of OH group by O and F terminations can significantly weaken the adsorption ability. Up to now, no experimental studies have been carried out for the adsorption of free heavy metal atoms on MXenes.

In short, first-principles studies have confirmed the excellent adsorption behaviors of MXenes for positively charged heavy metal ions and free non-ionic heavy metal atoms. For both types of heavy metal elements, surface terminations are found to significantly affect the adsorption behaviors, and generally speaking, –F group can reduce the adsorption ability of the MXenes. 

### 4.2. Radionuclide Elements Adsorption

With the increase in nuclear energy utilization, nuclear waste pollution is becoming a challenging environmental concern because the contamination of the radionuclides can be significant hazards even at trace amounts due to their long-term radiological and chemical toxicities. Regarding the charged state, radionuclide elements can exist in different forms, e.g., cationic UO^2+^, anionic TeO_4_^2−^, and neutral Xe. Up to now, only cationic UO^2+^ has been studied for the adsorption behaviors on MXenes.

**Figure 4:**
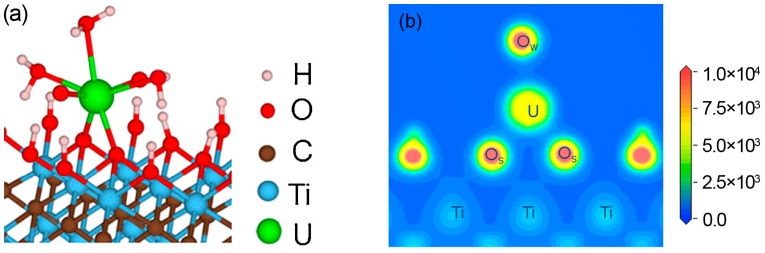
(**a**) Bidentate inner-sphere adsorption configuration of uranium ion on Ti_3_C_2_(OH)_2_ nanosheets; (**b**) Charge density distribution of the adsorption structure by density functional theory (DFT) simulations. Reprinted from Ref. [18] with permission. Copyright 2016 Elsevier B.V. (Color online).

Titanium carbide Ti_3_C_2_T_x_ is the first MXene material predicted to be an ideal adsorbent for radionuclide purification by Zhang et al. [25]. First-principles studies have shown that hydrated uranyl cation [UO_2_(H_2_O)_5_]^2+^ can be effectively adsorbed by Ti_3_C_2_(OH)_2_ MXene in aqueous solution. Uranyl preferentially adsorbs as a bidentate inner-sphere adsorption configuration coordinated to OH groups on the MXene surface. In this configuration, penta-coordinated uranyl species removes two coordinated water ligands and binds to two surface O atoms deprotonated from hydroxyl groups in activated Ti sites, forming a bidentate coordinated complex TiO_2_–UO_2_(H_2_O)_3_, as shown in Figure 4. Besides the U–O chemical bonds, hydrogen bonds between the two axial O atoms of the uranyl and the terminated H atoms on the MXene surface also contribute to the adsorption interactions. When coordinated by the anionic ligands such as OH^−^, Cl^−^ and NO_3_^−^, the uranyl species can also be strongly adsorbed by the Ti_3_C_2_(OH)_2_ MXene. Ab initio molecular dynamical calculations for the bidentate adsorption configuration of TiO_2_–UO_2_(H_2_O)_3_ in ambient water show that the water molecules do not have negative effects on the adsorption. Based on the stable adsorption configuration, the theoretical adsorption capacity is calculated to be as high as 595.3 mg/g for [UO_2_(H_2_O)_5_]^2+^ species.

Wang et al. experimentally confirmed the strong adsorption ability of V_2_CT_x_ MXene for uranyl species and analyzed the results with the first-principles theory [56]. The most energetically favorable adsorption configuration is the bidentate inner-sphere adsorption configuration, where penta-coordinated uranyl species removes two coordinated water ligands and forms chemical bonds with two surface O atoms deprotonated from hydroxyl groups, forming a VO_2_–UO_2_(H_2_O)_3_ complex, which is very similar to the situation of Ti_3_C_2_(OH)_2_. To further clarify the adsorption behaviors of uranyl on V_2_C MXene, the adsorption properties of V_2_C(OH)_2_ nanosheets for uranyl ions with different ligands in the general form [UO_2_(L_1_)*_x_*(L_2_)*_y_*(L_3_)*_z_*]^n^ (L_1_, L_2_ and L_3_ stand for H_2_O, OH and CO_3_) are studied by Zhang et al. [24]. The results show that all the uranyl species can bind strongly with V_2_C(OH)_2_ nanosheets with high adsorption energies greater than 3 eV. Among the studied uranyl species, aquouranyl [UO_2_(H_2_O)_5_]^2+^ bonds the strongest to the hydroxylated V_2_C nanosheet. It is also found that the terminated –F groups on V_2_C nanosheets could weaken the adsorption capability for uranyl ions, which is very similar to the results of heavy metal elements adsorption on MXenes.

In short, first-principles studies have confirmed the strong adsorption ability of hydroxylated MXenes for uranyl species UO_2_^2+^. The main adsorption mechanism is the chemical interaction between the U atom and two O atoms deprotonated from hydroxyl groups on the MXene surface. 

### 4.3. Gaseous Pollutants Adsorption

Industry discharged gaseous pollutants are becoming a critical issue, including toxic inorganic gases, and volatile organic compounds (VOCs). They can cause serious diseases to the respiratory system, and further other systems, of human beings. Several first-principles studies have been carried out on gaseous pollutants adsorption using MXenes, including NH_3_, SO_2_ and CO_2_. 

**Figure 5:**
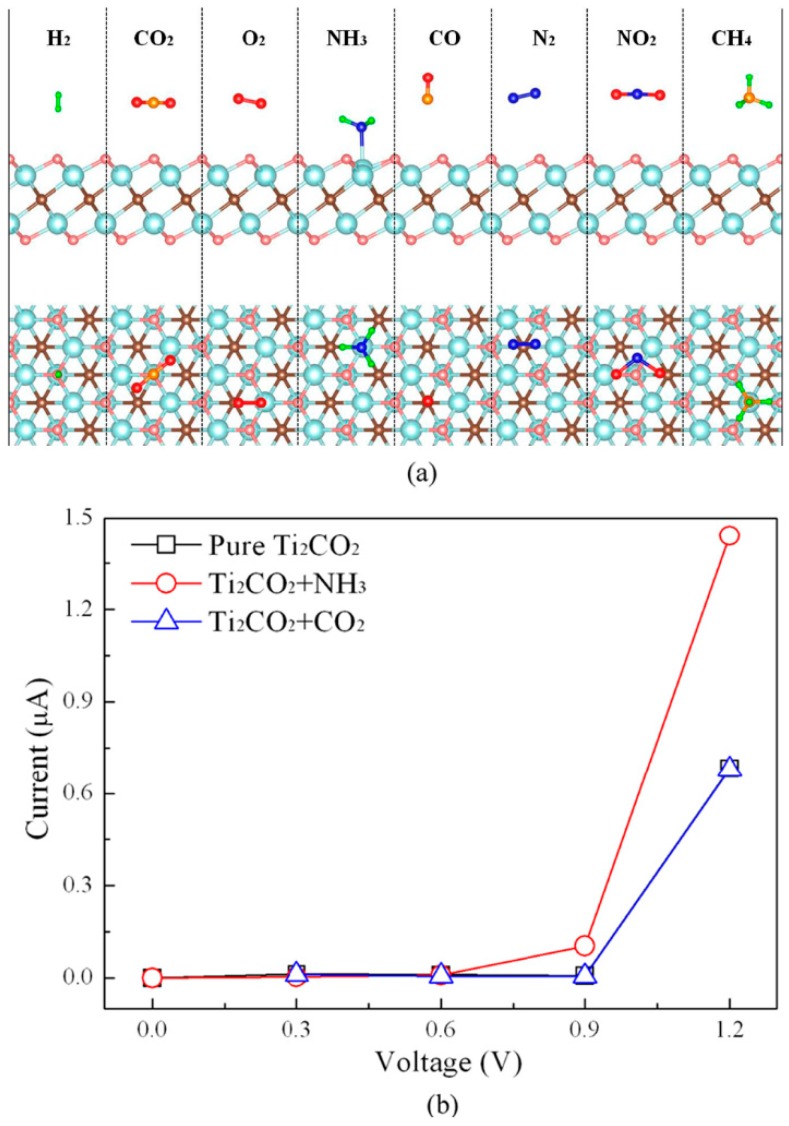
(**a**) A schematic illustration of Ti_2_CO_2_-based sensor for detecting NH_3_ molecule; (**b**) The current-voltage (I–V) relations before and after the adsorption of NH_3_ or CO_2_ molecule on monolayer Ti_2_CO_2_. Reprinted from Ref. [57] with permission. Copyright 2015 American Chemical Society. (Color online).

Yu et al. have investigated the adsorption behaviors of a series of gas molecules (NH_3_, H_2_, CH_4_, CO, CO_2_, N_2_, NO_2_ and O_2_) on monolayer Ti_2_CO_2_ MXene, and found only NH_3_ could be chemisorbed on the monolayer Ti_2_CO_2_ as compared with other gas molecules, as seen in Figure 5a [57]. Calculations show that N–Ti chemical interaction is the main adsorption mechanism. The adsorption energy of NH_3_ on Ti_2_CO_2_ is −0.37 eV. This intermediate energy implies that Ti_2_CO_2_ is a promising recyclable material for NH_3_ purification as it could easily release NH_3_. Furthermore, the electrical conductivity of Ti_2_CO_2_ is enhanced significantly after the adsorption of NH_3_, indicating Ti_2_CO_2_ could be a potential NH_3_ sensor with high sensitivity, as shown in Figure 5b. Additionally, the adsorption of NH_3_ on Ti_2_CO_2_ can be further enhanced by applying strain on the nanosheet. Xiao et al. further studied the adsorption behaviors of NH_3_ on a series of O-terminated semiconducting MXenes with the general form M_2_CO_2_ (M = Sc, Ti, Zr, and Hf) using first-principles simulations [58]. The results show that NH_3_ could be strongly adsorbed on all four M_2_CO_2_ MXenes with apparent charge transfer, which renders them the potential candidates as the NH_3_ sensor or capturer. In particular, the NH_3_ could be released by tuning the number of the electrons injected into M_2_CO_2_ MXenes. These results are very informative for practical applications of M_2_CO_2_ MXenes as NH_3_ sensors.

Going along with the similar thinking, toxic gaseous SO_2_ adsorption on O-terminated M_2_CO_2_ (M = Sc, Hf, Zr, and Ti) monolayers has also been studied based on first-principles calculations by Ma et al. [59]. It is found that Sc_2_CO_2_ is the most preferred monolayer for SO_2_ molecules adsorption with suitable adsorption strength (adsorption energy −0.646 eV) compared to other monolayers. The S–Sc chemical bonds are the main adsorption mechanism. Similar to the adsorption of NH_3_ on Ti_2_CO_2_, the adsorption strength of SO_2_ on Sc_2_CO_2_ can be further enhanced by applying strains on the nanosheet; and the conductivity of Sc_2_CO_2_ increases with the adsorption of SO_2_. It is noted that electric field has significant influence on the adsorption behaviors of SO_2_ on Sc_2_CO_2_: negative electric field facilitate the adsorption, while positive electric field weakens it (positive direction is the direction from the unadsorbed side to the adsorbed side). This characteristic is very meaningful for the applications of sensors or recycling use as adsorbent materials.

Morales-Garcia et al. first-principles calculated the adsorption behaviors of CO_2_ on unterminated M_2_C (M = Ti, Zr, Hf, V, Nb, Ta, Cr, Mo, W) MXenes and found that these bare MXenes can effectively adsorb CO_2_ even at low CO_2_ partial pressures and high temperatures, thus can act as very promising candidates for carbon dioxide capture, storage, and activation [60]. The adsorption mechanism involves complex interactions between CO_2_ molecules and the MXenes, and is dependent on the species of MXenes. Since CO_2_ is considered chemically inert, this result provides strong evidence that bare MXenes are very reactive for adsorbing pollutant species.

In short, O-terminated semiconducting M_2_CO_2_ MXenes exhibit reversible adsorption behaviors towards NH_3_ and SO_2_, and thus can act as gas sensors or adsorbents. Chemical bonds between the gas molecules and the M atoms of the MXenes are the main adsorption mechanism.

## 5. Summary and Outlook

First-principles calculations have shown that the MXenes, i.e., 2D transition metal carbides/nitrides display very encouraging performances in adsorptive remediation for various pollutants in polluted water and air, including heavy metal elements, radionuclides and gaseous pollutants. Different mechanisms contribute to different adsorption systems. For clarity, the MXene adsorbents and pollutant adsorbates, together with the main adsorption interactions, are summarized and displayed in Table 1. Experimental studies have confirmed some of the theoretical results. However, there are still several open questions that need to be addressed from first-principles view.

As discussed in above section, surficially bare MXenes are very reactive and able to adsorb a variety of pollutants species from water and air. At the same time, just because of this high reactivity, bare MXenes are very easy to react with ambient molecules, e.g., water and oxygen molecules [15,20,61,62]. These molecules may compete with the pollutant species in the adsorption process, and hinder the MXene materials in practical environmental remediation applications. Therefore, deeper studies of adsorption behaviors of pollutant species on bare MXenes, especially the competing behaviors between the pollutant species and ambient molecules should be conducted.

As we have shown, all the discussed charged heavy metal ions are cations. Since anions, e.g., Cr_2_O_7_^2−^, are also experimentally reported to be adsorbed by MXenes, deep first-principles studies of the adsorption behaviors of heavy metal anions on MXenes need to be carried out. Especially, regarding nuclides, only one species UO_2_^2+^ has been considered, and the adsorption behaviors of other nuclide cations and anions on MXenes are still blank and deserve investigations.

Most VOCs are chemically reactive and toxic to human beings, and experimental studies show that the physical properties of MXenes can be affected by adsorption of some VOC molecules, including ethanol, methanol and acetone, and thus can be used as VOC sensors [63]. Since the family of MXenes has a large number of members, adsorptions of different VOCs on different MXenes should be systematically studied based on first-principles method. 

In addition, although MXenes have been shown to be able to adsorb gaseous pollutants, e.g., NH_3_, SO_2_, and CO_2_ [57,58,59,60], there are no related experiments reported yet, which should be conducted to confirm their performances. After all, practical applications are the ultimate purpose of scientific studies.

## Figures and Tables

**Table 1:** A list of MXene adsorbents and pollutant adsorbates and their main adsorption interactions by first-principles studies.

MXene	Adsorbate	Main Adsorption Interaction	Reference
Ti_3_C_2_(OH)_2_	M_ad_(II) (M_ad_ = Pb, Cu, Zn, Pd, Cd)	M_ad_–O bonds	[47,48]
M_2_C(OH)_2_ (M = Ti, V, Cr, Nb, Mo, Hf, Ta) M_2_N(OH)_2_ (M = Sc, Ti, V, Cr, Zr, Nb, Mo, Hf, Ta)	Pb(II)	Pb–O bonds	[49]
M*_n_*_+1_C*_n_* (Ti_3_C_2_, V_2_C_1_, Ti_2_C_1_)	M_ad_ (M_ad_ = Pb, Cu)	M_ad_–M and M_ad_–C interactions	[30]
Ti_2_C(OH)_2_, Ti_3_C_2_(OH)_2_	Au	Au–OH bonds	[55]
Ti_3_C_2_(OH)_2_	U (IV)	U–O bonds	[25]
V_2_C(OH)_2_	U (IV)	U–O bonds	[24]
M_2_CO_2_ (M = Sc, Ti, Zr, and Hf)	NH_3_	N–M bonds	[57,58]
Sc_2_CO_2_	SO_2_	S–Sc bonds	[59]
M_2_C (M = Ti, Zr, Hf, V, Nb, Ta, Cr, Mo, W)	CO_2_	CO_2_–MXene complex interactions	[60]
